# Supplementary material for: Causal Relationship between Aging and Anorexia Nervosa: A White-Matter-Microstructure-Mediated Mendelian Randomization Analysis
Source: Biomedicines. 2024 Aug 16;12(8):1874. doi: 10.3390/biomedicines12081874 (PMC11351342; doi:10.3390/biomedicines12081874)
Supplement: Supplementary file 1 [file biomedicines-12-01874-s001.zip › Supplementary Figure/Figure S4/Singe SNP plot of MR analysis for TL to ubm-b-1875.pdf]

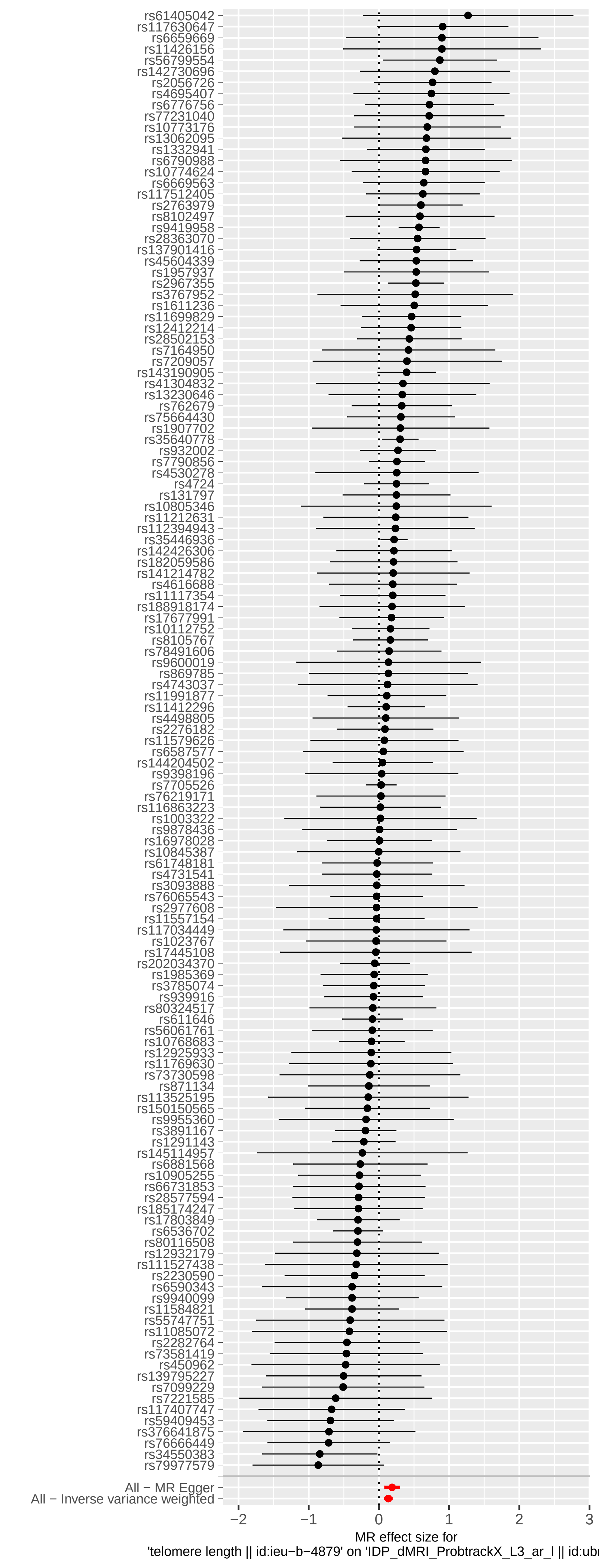
rs61405042
rs117630647
rs6659669
rs11426156
rs56799554
rs142730696
rs2056726
rs4695407
rs6776756
rs77231040
rs10773176
rs13062095
rs1332941
rs6790988
rs10774624
rs6669563
rs117512405
rs2763979
rs8102497
rs9419958
rs28363070
rs137901416
rs45604339
rs1957937
rs2967355
rs3767952
rs1611236
rs11699829
rs12412214
rs28502153
rs7164950
rs7209057
rs143190905
rs41304832
rs13230646
rs762679
rs75664430
rs1907702
rs35640778
rs932002
rs7790856
rs4530278
rs4724
rs131797
rs10805346
rs11212631
rs112394943
rs35446936
rs142426306
rs182059586
rs141214782
rs4616688
rs11117354
rs188918174
rs17677991
rs10112752
rs8105767
rs78491606
rs9600019
rs869785
rs4743037
rs11991877
rs11412296
rs4498805
rs2276182
rs11579626
rs6587577
rs144204502
rs9398196
rs7705526
rs76219171
rs116863223
rs1003322
rs9878436
rs16978028
rs10845387
rs61748181
rs4731541
rs3093888
rs76065543
rs2977608
rs11557154
rs117034449
rs1023767
rs17445108
rs202034370
rs1985369
rs3785074
rs939916
rs80324517
rs611646
rs56061761
rs10768683
rs12925933
rs11769630
rs73730598
rs871134
rs113525195
rs150150565
rs9955360
rs3891167
rs1291143
rs145114957
rs6881568
rs10905255
rs66731853
rs28577594
rs185174247
rs17803849
rs6536702
rs80116508
rs12932179
rs111527438
rs2230590
rs6590343
rs9940099
rs11584821
rs55747751
rs11085072
rs2282764
rs73581419
rs450962
rs139795227
rs7099229
rs7221585
rs117407747
rs59409453
rs376641875
rs76666449
rs34550383
rs79977579
All − MR Egger
All − Inverse variance weighted
−2
−1
0
1
2
3
MR effect size for
'telomere length || id:ieu−b−4879' on 'IDP_dMRI_ProbtrackX_L3_ar_l || id:ubm
